# Selections and Abstracts

**Published:** 1897-02

**Authors:** 


### *SELECTIONS AND ABSTRACTS.*

### The Autumnal Fevers of the Southern Atlantic States and Their Treatment.

This was the subject of an important paper by Dr. Bedford Brown, of Virginia, at the late Pan-American Medical Congress, from which we make following extracts (*Journal American Medical Association*):

Fever of a malarial origin is an annual visitant, from August to the middle of October, of all that vast section comprised within the Southern Atlantic and Gulf States, and also of a large portion of the interior, comprising the Middle, Western and Southern States. The large number of cases occurring within the vast area comprised within these borders, the distress of mind and body, the loss of time by sickness, the additional expense incurred, the impairment of health and the greatest of earthly evils, the mortality resulting, combine to render this one of the most important and interesting subjects in our profession, and how successfully to prevent malarial infection and to correct it after it enters the human system, become questions of paramount importance.

I find that this fever makes its appearance in our section about August 10 or 15 and continues to prevail until killing frost or freezing temperature begins, and then it as suddenly disappears as it appears. I find also among the profession and among the people that this fever in its simple, uncomplicated state, goes under the name of malarial fever, while in its complicated state and in its advanced stages, it is denominated typhoid fever. Hence, in all cases of malarial fever, characterized by symptoms of malignancy, as a dry tongue, delirium, jactitation, insomnia, tympanites, frequent pulse and general prostration are always miscalled typhoid, and by some typho-malarial fever. These misnomers and misunderstandings of the proper character of autumnal fever are very misleading in relation to our proper conception of the treatment and management of the disease.

In this fever which annually visits our section I do not believe that there is one case in twenty which is genuine typhoid or enteric fever, for these autumnal fevers, however they may in certain cases simulate true typhoid, disappear from our country at the first heavy frost, to return again at the next autumn. This is not the course, or a part of the history of true typhoid fever. Typhoid fever does not come in hot seasons and go in the cold, when frost and ice begin to make their appearance. Typhoid fever does not come when the temperature goes up to 80 or 90 degrees and disappear when it goes down to 32 degrees. But rather typhoid is a disease prevalent in low temperatures, in the late months of winter and early months of spring. On the contrary, malaria thrives, flourishes and grows in high temperatures and moist seasons, in flat, low localities. Typhoid fever has its favorite homes in high, cool localities in northern and mountainous sections.

The question of the origin of malaria, which is the true cause of our autumnal fevers, and of its peculiar nature, is now so well settled by scientific investigation it is unnecessary to discuss. The plasmodium malariæ of Laveran so clearly described by that careful investigator, its parasitic nature, its vegetable origin, its entrance into the circulation, its fastening itself on the red blood corpuscles as any other parasite, its growth and development in those corpuscles, its nourishment and sustenance on the material of the corpuscles, and finally its destruction of the red corpuscles, as any other parasite would do when fastened on an animal or vegetable body. This destruction of the red corpuscles of the blood explains many, if not all, of the pathologic changes resulting from malaria, as organic changes in the liver, spleen, blood and venous tissues. It explains the chills, the fevers, the congestions and irregularities of circulation of malarial fever.

The plasmodium malariæ of Laveran is probably the most certain diagnostic of malaria when found in the blood.

Another very interesting question in this connection is the channel through which the malarial plasmodium is carried and conveyed into the system, whether through the atmosphere or water, or both. Formerly it was the professional opinion that the air was the channel through which it was conveyed. At present the trend of professional opinion tends to the view that water is the principal channel. Experiments instituted by many observers in many localities go to show that water certainly is a common carrier of the malarial parasite. These experiments show that in malarial regions persons using surface water, as that of spring or wells or streams, are exceedingly subject to malarial poisoning, while the same persons when made to use water from the deep reservoirs of the earth, as for instance, from artesian wells, are entirely free of all malarial infection. In other words, that it is the water from the earth's surface saturated with the débris of vegetable decomposition that contains and carries the malarial parasite.

*Treatment.*—It is conceded by all authorities that malaria enters the human system either by means of the air we breathe or the water we drink, or through both of the channels. For many years it was the accepted opinion that the air was the only common carrier of malaria. More recently carefully conducted experiments go to prove that water is the true carrier of malaria. If this be true it is obvious that we have at hand a far greater command of the situation in instituting measures for the protection of the human system against malarial poisoning. Frost and malaria are deadly enemies. When water sinks in its temperature to 32 degrees F. all malarial germination and life ceases, and after that water in the most malarial districts may be imbibed with impunity. Our water supply, according to the latest and most reliable scientific experiments, is to be the field of future hygienic investigation and operation in regard to the question of malaria and its entrance into the human system. Formerly drainage, the clearing up of swamp lands, tillage and improvement of the soil were the only hope of those residing in malaial districts. Since the discovery that water, if not the chief, is a common carrier of malaria, another and a renewed hope has arisen for those who are yearly subject to malarial influences, and that hope lies in a supply of purified water. If this be trne, and all the evidences point in that direction, those who reside in the most deadly malarial districts may not despair, for by a little expense and not much labor, they can command the situation. It is very well established that malarial-infected water is confined to that on or near the surface of the earth. The deep reservoirs of the earth, which can only be penetrated by artesian wells, are free from malaria. But the expense of this resource renders it impracticable in many sections. But a resource available for the poorest and humblest in malarial regions is that of *sterilized water*, which has been subjected to the boiling point. But sterilized water, while its malarial parasites have been killed by the action of heat, nevertheless contains a certain amount of dead organic matter. From this objectionable element sterilized water can be made free by filtration.

Certain products of cinchona undoubtedly possess prophylactic power in addition to their curative properties. Quinin, its most important product, must be regarded, in addition to its multiplicity of medicinal properties, as an antidote to the malarial poison. And it has a claim to be ranked among the chemic antidotes. It arrests fungoid generation and growth by arresting all fermentative action in the blood. If the article quinin possesses the chemic property of accomplishing these objects it is entitled to be ranked among the antidotes for fungoid growth and fermentative action in the blood.

Its well known antipyretic powers in fevers are no doubt due to its remarkable antifermentative action.

Ten grains of the bisulphate taken in a glass of sherry wine before breakfast and previous to all exposure, when combined with the systematic use of sterilized water, will insure protection.

In the treatment of these diseases the important question arises, Can we by any known means modify the types of these fevers, by rendering them milder, and at the same time reduce their rate of mortality?

These are questions of paramount importance. I believe that by proper treatment these objects can be accomplished.

Quinin is the only certain and acknowledged antidote to the parasite of malaria.

But its efficiency as such depends absolutely upon the manner of its administration. Given according to rules it is a remedy of great precision. Given according to other methods it is entirely insufficient. There is much in the manner of giving remedies, and even the most valuable and potent may fail if given without proper method or system.

Forty years ago it was the custom to give quinin in malarial fever in doses of one grain every hour, or two grains every two hours, or three grains every three hours. Well do I remember the utter inefficiency of the remedy in modifying the type or reducing the rate of mortality. By this method the system failed to get sufficient of the antidote to destroy the parasite. In giving this antidote it becomes somewhat a question of mathematical calculation. We must gauge the quantity of our antidote to the amount of parasite in the system and the gravity of the case. Not only this, but the remedy should be given in large doses at longer intervals, rather than in small doses at shorter intervals. Thirty grains given in ten-grain doses three times a day is far more efficient in the remittent forms of fever than the same quantity given in two-grain doses every two hours. Twelve-grain doses morning and evening act more decidedly than twenty-four grains divided into broken doses every two hours. In decided forms of fever quinin should never be given in small doses, however often repeated. I have repeatedly seen cases in which one or two grains were given every one or two hours, without the least effect, when the same quantity per diem given in three equal parts three times a day changed the entire aspect of the case promptly.

For many years I have taken every opportunity to experiment with quinin in malarial fevers with a view of ascertaining in each form and stage of fever the quantity of quinin that was necessary to act as a destructive antidote to the malarial parasite.

I found by experiment that in the treatment of intermittent fever fifty grains of quinin given within thirty-six hours preceding the chill, was the maximum quantity required to arrest the disease. Thirty grains given within twenty-four hours preceding the chill in a majority of cases would arrest it, but not invariably. I found that five grains of acetanilid given just before the chill invariably modified the chill and resulting fever. In regard to chronic chill and fever, I found ten grains of quinin given in sherry wine before breakfast and Warburg's tincture in full doses after dinner and supper, almost invariably acted as preventives.

Quinin should never be given in pill form, as they are slow to dissolve and frequently do not dissolve in the stomach at all. It is preferable to give it in solution or powder as the bisulphate, or lastly in fresh capsules. I am satisfied that a certain proportion of cases are lost because of the insolubility of pills. I have repeatedly seen in my practice cases grow worse daily under the use of the quinin pill, that improved rapidly when the solution or powder was substituted. Then again in cases of great emergency, where we desire prompt action, in my experience, the bisulphate is the most certain form in which it can be given.

In the application of this remedy as an antidote to the malarial plasmodium, we must be governed by fixed laws and practical rules, or failure will be the result.

It is certainly true that we have all grades and types of malarial disease from the mildest to the most malignant, and we must adapt our measures to these different grades or we cannot have success. I found in my experience in the past thirty years, in about one hundred and seventy-five cases of malarial fever beginning suddenly, with a decided chill and followed by a temperature running up rapidly to 105 or 106 degrees, with intense neuralgic pains, sixty grains of quinin per day of twenty-four hours given in divided doses of ten grains every four hours would invariably arrest the attack in seventy-two hours and frequently less time. Thirty grains per day would prolong the attack to five or six days, especially if given in three grain doses every two hours. Twenty grains per day would prolong the attack between one and two weeks, and fifteen per day would prolong it from three to four weeks.

These facts teach us the important lesson, that to obtain the full antidotal effects of quinin, we must saturate the system with the remedial agent promptly in quantities sufficient to kill every malarial germ in the system. Otherwise if a single germ is left in a living state it becomes the nucleus for rapid germination and multiplication, and our work must be done over again.

The prolonged form of malarial fever, if neglected or improperly treated, is certain about the third or fourth week to pass into the adynamic stage.

The questions arising in treating this form of fever are, whether its type can be modified, its progress curtailed, and the adynamic stage be averted.

I can with the utmost certainty answer this question in the affirmative. But in our treatment we have persistent disease to contend with, and our treatment must be systematic, constant and active, and as sure as we lapse into an expectant method, or relax our efforts, the progress of the case gains on us every hour and it will pass into the adynamic stage, there will be increase of temperature, of pulse rate, and the rhythm of fever will disappear and it will assume a continued form, and then the stage of delirium appears, with all other toxic symptoms. If we expect success in our treatment the antidotal treatment must be commenced from the earliest stage, and never relaxed to the end. Twenty grains of quinin divided in three equal parts given three times a day will maintain the case in its simple type, keep the temperature down to 101 degrees in the morning and 102 degrees at evening, prevent typhoid symptoms, or other complications and finish up the case about the third or fourth week, but 30 grains per day will do much better.

It is surprising how the system comes to tolerate these full doses of quinin when long continued and which are followed by no evil results. I have given continuously for a month 10 grains three times a day, and after the first few days all unpleasant effects on the nervous system would cease, where there would be a complete state of tolerance, and the remedy would act as a pleasant sedative tonic and antipyretic.

---

### EXPERIMENTS WITH SERUM FROM SYPHILITICS.*

In *La Médicine Moderne* of November 28, 1896, is an interesting paper by G. Marēchal describing some investigations made by him into the artificial production of immunity against syphilis, and an account of a micro-organism which he thinks to be the specific cause of the disease. The paper is too long to be reproduced *in toto*, but we give its substance in the extracts presented below.

" Of all the infectious diseases, syphilis is the one whose patho-

*Translation by Dr. Chas. M. Blackford, Atlanta.

genesis has remained the most obscure, because it is essentially a human disease and because we know neither its specific micro-organism nor its lesions in animals. We have studied it by auto-inoculations on ourselves, to the end that we might be immune to syphilis.

"To realize this immunity we had recourse to injections of syphilitic serum taken from seven patients.

"After injecting the serum from a syphilitic subject whose affection was of about twenty-five years' standing, we inoculated ourselves with that of two other patients who had contracted the disease about five years before, then of a person whose indurated chancre had made its appearance only about three weeks and finally from one of three months. The last injections that we gave ourselves were from a patient infected with syphilis for seven and one-half months, and who had ulcers scattered over his back and legs.

"On the third day, our attention was attracted to a phenomenon which has enabled us to measure approximately the virulence of the serum injected—the injection of 2 c. c. of serum from a patient with general paralysis of syphilitic origin produced in about an hour swelling of a gland in connection with the lymphatic region in which was the seat of the inoculation. We set up this glandular reaction every time we injected a virulent serum taken from a syphilitic subject. On the other hand, when we injected normal human serum, an adenitis was never produced. Moreover, this glandular reaction manifested itself after a somewhat shorter time, persisted longer, and was more soft and painful as the serum was more virulent. But when this virulence was raised above a certain degree, the adenitis had a tendency to become hard, and would not subside for three or four hours instead of one hour after the inoculation. Persuaded that this glandular tumefaction coincided with an afflux of phagocytes into the gland, that the dispersion of the tumefaction indicated that the phagocytes had digested both the microbes and the toxins, and that they are carried away by the current of the circulation, we thought that our degree of immunization rose progressively, and that after each inoculation we were more protected from syphilis as the adenitis disappeared.

"The injection of a serum obtained from a patient who had been infected with syphilis for seven months and who presented syphilitic ulcers scattered over his body, set up in us after a dose of 2 c. c., an axillary adenitis which persisted for about two days; we caused it to disappear by an injection of serum from a horse. Thereafter when an adenitis took too long a time to subside we had recourse to the serum of a horse.

"But this normal curative serum made us rash: the injections of the seven months' syphilitic serum, repeated and in larger doses, practiced on the right buttock and in the right sub-umbilical region, brought on a hard and indolent adenitis that the serum from the horse could not make disperse. Our serum, obtained from bleeding the arm, caused the gland noticeably to diminish, but without definitely triumphing over the swelling or the glandular induration.

"Seven days after these last inoculations of the seven months' syphilitic serum, a hard button appeared on the right buttock: we caused it to disappear by one injection of the horse's serum in the very center of the button; a second indurated button appeared some days after on the right buttock and disappeared same way under the influence of the horse's serum; a third indurated button manifested itself in the right sub-umbilical region, and ulcerated in spite of injections of the horse's serum; the indurated chancre was formed.

"We now anxiously watched ourselves until the appearance of the roseola, which was localized in Scarpa's triangle. The inoculation with the seven months' syphilitic serum had taken place on the 21st of January; the indurated chancre had appeared on the 28th of January; the localized roseola did not commence until the 21st of March; the whole region of Scarpa's triangle was discolored from a pigmentary point of view; the surface of the skin presented a peach-blossom tint, which grew gradually fainter from the base to the apex of the triangle; this roseola corresponded with an adenitis that also grew less marked as it involved glands nearer the apex. After this miniature manifestation of secondary syphilis, we observed no generalized roseola, no generalized adenitis, no alopecia in thin spots, no syphilides, no nocturnal headaches and no albuminuria. The urine was normal, except that the amount was reduced to 12 grammes in twenty-four hours for two or three months. The blood was normal, the injection of our serum, intended to set up a glandular tumefaction, produced on the contrary the rapid disappearance of the adenitis provoked by the injection of the syphilitic serum. To sum up from a clinical stand point, syphilis is essentially glandular; the first period, mono- or pauciglandular, may in turn be divided into three phases: the indurated gland, the indurated button, and the indurated chancre. The second period, multiglandular, reveals itself as a generalized roseola.

"As we have never observed the glandular reaction that is provoked by the inoculation of a virulent syphilitic serum, excited by a normal human serum, we consider this phenomenon as the pathognomonic sign of the virulence of the blood of the syphilitic subject. As the idea of virulence implies a micro-organic origin, we have sought to discover if the specific microbe of syphilis exists in the blood of those afflicted with the disease."

The author then describes a micro-organism that he found in the blood of a patient. He says: "In an unstained preparation, the bacilli took the form of little clubs almost broader than long, rounded at the extremities and much constricted in the middle; one would say they were two cocci put side by side but joined by a very narrow segment: one can therefore call them bacilli pseudo-diplococci. They are immotile.

"This bacillus grows on human blood serum, acetic fluid, gelatine and urine, in which last the growth is peculiar and, as claimed by the author, characteristic. It grows best on a mixture of gelatinized serum of a horse mixed with one-third human serum."

M. Maréchal claims that the same reactions follow injections of this bacillus as of syphilitic serum.

---

### NECESSITY OF STRICT DIETING IN SKIN DISEASES.

Brocq (*Journal de Méd. et de Chir.*, March, 1896) insists that not only are there certain kinds of food which provoke immediate eruptions of the skin--such as sea fish, oysters, preserved meats, game, pork, cheese (which very often causes acne and folliculitis), certain acid fruits, strawberries, raspberries, gooseberries, cabbage, nuts, etc.

—but that others act with a more delayed effect. The noxious effects of alcholic liquors, beer, wine, coffee, and tea are not generally admitted, yet they often cause urticaria. These ill-effects are supposed to be caused by a veritable intoxication of the system, which they produce by their absorption, or by provoking some trouble of the stomach and intestines, and consequently influencing the whole organism. These eruptions can also occur from the elimination of irritant elements through the skin; in this case not only the food is concerned, but also the manner of preparing it. Certain persons cannot eat butter, especially if it is adulterated, and fat, oil, spices and under-done meat produce ill-effects upon others. Moreover, individual idiosyncrasies must be considered. These are not always the same during life, as sometimes we meet people who can at a certain time support a given substance, which, later, without any ascertainable reason, may cause an eruption.

It is generally easy to obtain from a patient a promise to abstain from food that produces ill-effects, no matter how pleasing it may be in taste, if these effects are immediate: but when the effects become apparent only after the lapse of considerable time, as in gout or rheumatism, it is hard to obtain such a promise. Yet it is reasonably certain that effects upon the skin are often produced as remote in time from the ingestion of the deleterious article of food as in the case of these two named diseases, although unfortunately for science, this remote effect is denied by the majority of dermatologists.

Observation has shown that if alimentation is defective either through excess, want, bad qualities of the ingesta or non-adaptation of food to circumstances and climate, either the food is badly digested, badly absorbed, and especially badly elaborated in the body, and the divers excretory functions become insufficient in their workings. In such cases, little by little, more or less toxic excremental matters are accumulated in the body, impressing upon the organism a seal of vital decadence and morbid vulnerability. It is known that individuals leading sedentary lives who eat heartily of substantial food, and in addition are moderate drinkers, or who are fond of tea or coffee, are subject to gout or rheumatism. If this is granted, why should the skin alone possess an immunity to the slow toxic effects of the gradual accumulation of excremental poisons? Frequently, in people of either hereditary or acquired gouty diathesis, are found cases of bullous or erysipelatous eczema and limited neurodermites, alternating with visceral and articular manifestations. Chronic alimentary intoxication must in all cases be considered the most important cause of the cutaneous symptoms.

The same considerations can be applied to many cases of nervous excitability, alimentation playing a more important part than is generally supposed possible. In a series of cases extending over a number of years, Dr. Brocq found excessive coffee-drinking to be the cause of many pruriginous dermatoses. In many of these cases coffee took the place of food to a large extent, and the patients were extremely thin with trembling of the extremities. Nervous affections of the skin, with or without eczema, were especially numerous.

It then seems logical to advise arthritic people to observe a strict diet if they wish to avoid annoying eruptive attacks. They should particularly abstain from coffee, liquors, wine, beer, dark meats, and acid vegetables and fruits. Persons affected with acne must avoid salty cheese, preserved meats and fish. These precautions must be particularly observed when an attack is imminent because of development of the morbid predisposition. The articles of food mentioned can play a rôle of accidental cause under these conditions and have an immediate pathological effect.—*Med. and Surg. Reporter. The Cincinnati Lancet-Clinic.*

---

### PRACTICAL POINTS IN ELECTRO-THERAPEUTICS.

General practitioners desiring to use electricity in their practice, but possessing practically no knowledge of the electro-therapeutics, will do well to read, study and learn the following points:

The faradic or interrupted current cures disease mainly through physiological and mechanical processes, producing no appreciable chemical effect in the tissues.

In each faradic battery there should be two coils—primary and secondary.

The current from the primary coil has greater quantity and produces greater mechanical effects, but lacks penetrating power and is more useful in superficial troubles, particularly where mechanical efforts are desired.

It is also of service in internal treatments for many vaginal and uterine diseases.

In the primary current there is some difference in polarity, the positive pole producing greater sedation and the negative pole greater stimulation.

The secondary coil produces a current having much greater penetrating power than the primary current.

It will more easily overcome resistance on account of its greater tension, and is to be preferred to the primary current in treating deeply seated conditions.

There is no appreciable difference in polarity in the current from the secondary coil.

The current from the secondary coil is much more pleasant in its effect, particularly to nervous subjects.

In the direct galvanic current there are no interruptions to the current as in the physiological and mechanical effects.

There is no sensation to the current beyond a feeling of numbness and burning beneath the electrodes.

In general terms, it may be stated that the faradic current is to be preferred in muscular troubles and the galvanic in nervous diseases.

The galvanic current will produce a greater degree of sedation or stimulation.

The galvanic current should be used to stimulate the absorbents, to remove effusions, morbid growths, callosities, tumors, facial blemishes, superfluous hairs, etc. In these conditions the faradic current is practically powerless.

The positive pole of the galvanic current is much more sedative and the negative pole much more irritating and stimulating.

Electrolysis and cataphoresis can only be used with the galvanic current.

To produce the most pronounced sedative effect and allay irritability make stabile applications of the positive pole, galvanic current.

To produce the most marked irritation, use the negative pole of the galvanic battery, or reverse the polarity frequently.

Electricity has a decidedly refreshing and soothing effect upon the entire nervous system, and is of great service in nearly all nervous diseases.

Either faradic or galvanic current will equalize the circulation, increase the flow of blood to the surface and extremities, and improve nutrition and assimilation.—*Electro-Therapeutist.*

---

### TREATMENT OF ACUTE SORE THROAT OF NON-DIPHTHERITIC TYPE.

Lyon, in the *Revue de Thérapeutique Médico-Chirurgical* of May 15, 1896, recommends the following treatment in sore throat not dependent upon a specific diphtheritic infection: Internally small doses of one of the salts of quinine or of antipyrin, either in cachet or in solution. Generally cachets are swallowed with too great difficulty by children:

℞ Antipyrin ........................................15 to 45 grains.
Water ........................................3½ ounces.
Syrup........................................1 ounce.
A teaspoonful three times a day.

Or,

℞ Neutral sulphate of quinine........................7 to 15 grains.
Syrup........................................6 drachms.
Syrup of codeine........................................6 drachms.
Distilled water........................................3½ ounces.
Teaspoonful at three- or four-hour intervals.

Sometimes, if there is a rheumatic taint in the case, benzoate of sodium and salol are useful. The dose of the salol should be large, and it is claimed that it will relieve the pain, reduce the temperature, and exercise a general anesthetic influence which is of value. Where the sore throat is accompanied by marked systemic disturbance, particularly with disorder of the digestive tube, it may be well to give benzo-naphthol, and, as a tonic, cinchona or alcohol.

The local treatment he recommends is to allow a small piece of ice to melt in the mouth, or to use a gargle of cocaine as follows:

℞ Boric acid........................................... ½ ounce.

Hydrochlorate of cocaine ........................ 7 grains.

Glycerin .................................................. 1½ ounces.

Water ...................................................... 10 ounces.

Or the tonsils, if they are inflamed, may be painted with glycerite of menthol and cocaine, such as hydrochlorate of cocaine 4 grains, menthol 15 grains, olive oil 1 ounce.

Compresses soaked in hot water may also be put about the throat and covered by gauze and rubber dam.

Locally antisepsis may be maintained by the use of gargles which may contain boric acid, salicylic acid, carbolic acid, or guaiacol.

The gargles which he thinks are most useful in ordinary sore throat are as follows:

℞ Salicylic acid ........................................ ½ to 1 drachm.

Borax ...................................................... ½ to 1 drachm.

Honey ...................................................... 1 ounce.

Distilled water ......................................... 8 ounces.

Or,

℞ Borax ...................................................... 2½ drachms.
Hot water ................................................ 6 ounces.
After solution, add:
Tincture of myrrh ..................................... 1 drachm.
Syrup of mulberries ................................. 1 ounce.

As a local application by means of painting, a mixture of equal parts of guaiacol and glycerin may also be employed. The guaiacol acts as a local anesthetic. After the patient is convalescent, care must be taken that relapse does not take place. The antiseptic gargles should be continued, and the following prescription may be given internally to aid this purpose:

℞ Crystalline carbolic acid ......................... 1 drachm.
Alcohol .................................................... 6 drachms.
Essence of peppermint ............................ 15 grains.

Eight to ten drops of this may be given in a glass of water.—*Therapeutic Gazette.*

---

### THE X-RAY IN SURGERY.

At the late Pan-American Medical Congress, in Mexico, Dr. Carl Beck, of New York, read a paper on this subject. He stated that the rays had been successfully used in the detection of splinters of metal and glass, bullets, needles, etc., which can be easily photographed or seen by the fluoroscope. It is also easy to perceive topographic-anatomical representations of the skeleton, the position of the fetus in utero, deficiencies of bones, deformities, dislocations, callous formations and complications in fractures, and such diseases of joints as pseudarthrosis, ankylosis, hypo-peri and exostosis, and ostitis and osteomalacic processes are representable. The area of ossification in rachitis, the dentation line in hereditary syphilis, caries of bones, tissue change in arthritis, osteophytes, calcination, ossification of cartilage, and tumors of the bones can be recognized. The case in which Koenig recognized a sarcoma of the tibia by the light mass of a lobular structure contrasting with the dark diaphysis of the tibia was cited. Suspected green-stick fractures, as well as the manner in which it is treated, may be demonstrated by reproducing the limb, together with the bandages, splints, etc. This Roentgen picture may thus become a valuable document in a medico-legal way. Neusser was quoted as having proved the phosphates as well as other calculi in the urine by the rays. The great obstacle in the diagnosis being the situation of the bladder in the bony pelvis, which prevents the permeation of the rays. It is claimed that renal calculi, as well as gall-stones could be diagnosed, but the essayist has been unable to demonstrate this. He has photographed the skull, and recognized foreign bodies in it, as well as sklerosis of the arteries, while calcified deposits in the lung, the remainder of old, healed tuberculous ulcers, could be plainly seen. The organs, which are kept moving by the respiratory and circulatory acts, can be seen with the aid of a fluoroscope, but cannot be photographed. The only method of recognizing effusions is by finding a translucent space between two organs, and an abscess might thus be suspected. Regarding the curative effect of the rays in zymotic diseases, Dr. Beck stated that the vitality of pathogenic bacteria is not impaired by the rays, even if the exposure lasts for several hours.—*Ex.*

---

### PURULENT DISCHARGES FROM THE MIDDLE EAR.

This is a subject which is deserving of much more attention from general practitioners than it usually receives. Such cases, if neglected, are not only a menace to hearing but even to life itself, particularly in children, in whom the part of the temporal bone between the aural cavity and the brain is often as thin as tissue paper. The treatment of chronic suppurative otitis media is discussed in a practical manner by Norton L. Wilson in the *New York Medical Journal* of March 28. The first point, he says, is the thorough removal of all secretions and débris. This is usually accomplished by syringing with slight force through the external meatus an aqueous solution of common salt or boric acid, twelve to sixteen ounces three or four times a day. If the discharge is offensive, Dr. Norton uses a five per cent. solution of permanganate of potassium, a one-half per cent. solution of carbolic acid, or even corrosive sublimate $\frac{1}{10000}$. The latter should not be intrusted to the patient himself. After cleansing the cavity in this manner the writer instills into the ear a twenty-five per cent. solution of neutral hydrogen peroxide and forces out any remaining pus with the Politzer bag. If this procedure fails, inflation may be performed with the Eustachian catheter, and any loose material in the cavity of the tympanum can be cleared out with a medicine dropper. Where syringing is contraindicated on account of giddiness and other unpleasant effects, we may try mopping out the middle ear with absorbent cotton and dropping in a little alcohol. If the perforation is too small for thorough cleansing of the cavity, it is better to enlarge the opening. All débris, exudations, polypi, granulations and necrosed bone must be removed, the general health looked after, and above all any nasopharyngeal troubles should receive due attention.—*Denver Medical Times.*

---

### INFECTIOUS VULVO-VAGINITIS AND PURULENT OPHTHALMIA IN CHILDREN.

Dr. Henry B. Sheffield contributes an interesting article on the above subject to a recent number of the *American Medico-Surgical Bulletin.* His conclusions, based on a study of sixty-five cases, are as follows:

1. Infectious vulvo-vaginitis in children is of gonorrheal nature; the diplococcus present in the purulent discharge is invariably identical with that of Neisser, decolorizing by Gram's method.

2. The infection can be conveyed through common privies, baths, beds, clothing, etc.

3. The symptoms accompanying the disease are far less severe than those described in most text-books.

4. Most of the complications are preventable.

5. The value of boric acid or mild silver nitrate solutions as prophylactics of purulent ophthalmia is very doubtful.

6. Silver nitrate in strong solution is a reliable abortive of purulent ophthalmia, if used in the very earliest stage.

7. The mere presence of gonorrheal discharge in a small girl, without injury to the genitalia, does not prove that rape has been attempted.

8. Physicians in charge of asylums, or similar institutions should be on their guard not to admit girls with vaginal discharge, unless they can convince themselves that this is not of gonorrheal origin.

9. The subject in question deserves a more careful study by the gynecologist, pediatricist, as well as by the general practitioner and medical jurist; and by their united observation we should in the near future be enabled to dispel any and all doubt as to the real nature of infectious vulvo-vaginitis in children—*Clinical Recorder.*

---

THE CUTANEOUS MANIFESTATIONS OF DIABETES. Prince A Morrow. (*Medical Record,* Vol. XLIX., p. 508.)

Affections of the skin occurring in diabetes may be divided into two classes, those which are of definitely diabetic origin (*diabètides* of French writers), and those which occur accidentally. In the former class are included pruritus, diabetic erythema, eczema, balanoposthitis, furuncles, carbuncles, gangrene, and xanthoma diabeticorum. The sebaceous and sweat glands are disordered, the secretion being generally diminished (asteatotis, anidrosis), but in a few cases increased (hyperdrosis). Allied to this defect is the falling out of the hair often observed, the nails, too, are often cast off, owing to the suppuration of the matrix. The eruptive disorders, erythema and eczema, are preceded by pruritus, and are probably in part due to traumatism (scratching). Eczema vulvæ is characterized by great thickening of the mucous membrane, a dark-red color, and dry, glazed appearance, and a similar thickening and infiltration is seen in diabetic balanoposthitis and its resulting phimosis. Diabetic gangrene may be either spontaneous or the result of slight trauma. It is usually moist, and occurs mainly in the old and obese, and the vessels are, as a rule, found to be pervious. Xanthoma diabeticorum has been recorded altogether in twenty-one cases, and its casual relation to the disease is shown by its involution as the glycosuria diminishes, and its reappearance as the sugar returns. The nodules are firm and of inflammatory nature, and are accompanied by subjective sensations of heat and itching. Of the accidental skin lesions in diabetes may be mentioned psoriasis, which has often been observed; dermatitis herpetiformis, of which four cases occurring in diabetics have been recorded (Winfield, *Jour. Cut. and Gen. Urin. Dis.*, November, 1893), and a peculiar bronzing of the skin described by v. Harndt (*Wiener Med. Blätt.*, 1896, p. 101).—WM. CECIL BOSANQUET.

---

### A CASE OF HERPES ZOSTER OF THE RIGHT UPPER EXTREMITY (*Clinic Atlanta Medical College*).

This case of what was formerly called "shingles" affected a light mulatto girl of about ten years of age. There was a pale red patch with fine vesicles at edge of thenar eminence; a red, well-defined one, studded with shot-sized vesico-pustules, on skin over depression between extensors of thumb and index-finger; another on radial side of forearm, and a few ill-defined above the elbow. The disease is characterized by the occurrence of neuralgic pains in the points of eruption, often preceding the skin lesions, or pains referred to the spinal center. In this case the eruption appeared over the terminal filaments, chiefly of the radial and external cutaneous nerves. Lesions of the cord have been found in some of these cases. The disease is considered self-limited, the duration being ten days to two weeks. Ichthyol, three to four per cent., in collodion, is a good application. Morphine-collodion, local galvanism, etc., have been employed. M. B. H.

### OXYGEN GAS IN NOSE AND EAR DISEASES.

Dr. George Stoker, of London, has called attention in a late paper before the American Laryngological Association, to the use of oxygen in ozena (syphilitic and chlorotic) and in chronic suppurative otitis media. He was induced to try it for these conditions from observing its curative effects upon wounds and suppurative surfaces in other parts of the body. The oxygen is contained in a mackintosh bag with two taps, one for inlet and one for outlet; it is combined with an equal part of purified air and the bag contains one cubic foot of the mixed gas. This amount is sufficient for about six hours' treatment. The nose piece is passed into one nostril and the other is plugged with cotton. In ear cases the terminal piece is placed in the external auditory canal. The oxygen should be allowed to pass into the nose or ear from three to six hours daily, with intervals of rest. The author reported four cases of ozena and suppurative middle ear disease treated and cured by this method. See *New York Medical Journal,* August 29, 1896.

---

### FORMALIN AN APPROXIMATE SPECIFIC FOR RINGWORM.

An interesting editorial note has appeared in *Guy's Hospital Gazette* calling attention to a recent paper by Mr. Alfred Salter, on the treatment of ringworm by formic aldehyde, or formalin. This treatment is now so well known in Guy's, and has had such a conspicuous success, that it should be part of the ordinary practice of every old Guy's man. There seems no doubt that it is the almost specific treatment for the disease, especially in obstinate and hitherto incurable cases. And yet this discovery arose from the annoying fact that the inventor's cultivations of the ringworm microbe were all killed one night through his having left the stopper out of the formalin bottle. So do fates at times turn good out of evil. *The Therapist* (London), November 15.—*Journal American Medical Association,* January 2, 1897.

